# Estimation of Intradialytic Blood Volume Reduction Using Hemoglobin Changes: A Prospective Validation Study of a Pragmatic Clinical Tool

**DOI:** 10.3390/jcm15114323

**Published:** 2026-06-03

**Authors:** Nomy Levin Iaina, Muhamed Osman, Fadi Garzuzi, Sergey Bellov, Arie Feldman

**Affiliations:** 1 Department of Nephrology and Hypertension, Barzilai University Medical Center, Ashkelon 7830604, Israel; mohammadu@bmc.gov.il; 2 School of Medicine, Faculty of Health Sciences, Ben Gurion University of the Negev, Beer Sheva 8410501, Israel; 3 Internal Medicine Ward B, Barzilai University Medical Center, Ashkelon 7830604, Israel; fadigarzuzi@gmail.com (F.G.); bellov@gmail.com (S.B.); aryef@bmc.gov.il (A.F.)

**Keywords:** hemodialysis, blood volume reduction, hemoconcentration, volume status, intradialytic hypotension

## Abstract

**Background/Objectives:** Accurate assessment of intradialytic blood volume (BV) changes is important for optimizing fluid management in hemodialysis, but continuous BV monitoring is not universally available. Hemoglobin changes reflect hemoconcentration and may provide a simple surrogate for estimating BV reduction. We prospectively evaluated a hemoglobin-based method for estimating intradialytic BV reduction compared with machine-based BV monitoring. **Methods:** In this prospective single-center observational study, 187 hemodialysis sessions with complete paired measurements were analyzed. Formula-based BV reduction was calculated from pre- and post-dialysis hemoglobin values and compared with machine-measured BV reduction. Agreement was assessed using Pearson correlation, predefined absolute-difference thresholds, and Bland–Altman analysis. Exploratory receiver operating characteristic analyses evaluated the ability of formula-based estimates to identify sessions with larger machine-measured BV reductions. **Results:** Formula-based and machine-measured BV reduction demonstrated a moderate-to-strong correlation (r = 0.645). The predefined pragmatic agreement criterion of ≥70% of measurements within ±5% was met, with 77.5% of measurements within this range. Bland–Altman analysis demonstrated a small mean bias of −1.5%, with 95% limits of agreement from −12.4% to 9.3%. Exploratory classification performance was favorable across machine-defined BV reduction thresholds, with area under curve (AUC) values ranging from 0.84 to 0.87. At the ≥8% threshold, sensitivity was 72%, specificity 85%, positive predictive value 83%, and negative predictive value 74%. Linear regression showed that 42% of variability in machine-measured BV reduction was explained by the formula-based estimate. **Conclusions:** A hemoglobin-based approach provides a simple approximation of intradialytic BV reduction. Although not interchangeable with continuous monitoring, it may support post-session assessment and longitudinal evaluation of intradialytic hemodynamic tolerance.

## 1. Introduction

Assessment of intravascular volume status is a fundamental challenge in clinical medicine and is particularly critical in patients undergoing hemodialysis. Both hypovolemia and fluid overload are associated with significant morbidity and mortality, including impaired tissue perfusion, cardiovascular complications, and increased risk of death [1,2,3,4,5,6]. Despite its clinical importance, accurate evaluation of volume status remains difficult, as commonly used clinical, laboratory, and imaging parameters lack sufficient sensitivity and specificity to reliably quantify intravascular volume changes [7,8,9,10,11,12,13].

In patients with end-stage kidney disease (ESKD), fluid management represents a central component of care. During hemodialysis, several liters of fluid may be removed through ultrafiltration to achieve euvolemia and maintain an appropriate “dry weight” [14]. However, determination of the optimal dry weight is imprecise and largely based on clinical judgment. Excessive ultrafiltration may lead to intradialytic hypotension and hypovolemic complications, whereas insufficient fluid removal contributes to chronic fluid overload and adverse cardiovascular outcomes [4,5,6]. Therefore, accurate assessment of intradialytic blood volume changes is essential for optimizing dialysis therapy.

Technologies for continuous monitoring of relative blood volume during hemodialysis have been developed and are integrated into some dialysis machines. These systems, typically based on optical measurement of hemoglobin concentration within the extracorporeal circuit, allow noninvasive real-time estimation of intradialytic blood volume changes and have been validated in both in vitro and in vivo settings [15,16].

Although integrated relative blood volume monitoring is available on many modern dialysis platforms, its use remains inconsistent across dialysis units, machines, and treatment sessions. Availability may be limited in centers using heterogeneous dialysis equipment, older machines, or devices without activated blood volume monitoring modules. In addition, even when the technology is available, it is not necessarily incorporated into routine clinical workflows for every hemodialysis treatment. These practical limitations create a need for complementary approaches that rely on routinely collected clinical data and can provide accessible post-session assessment of intradialytic blood volume change.

Unlike integrated blood volume monitoring systems that provide continuous real-time intradialytic data, the present approach is intended as a pragmatic post-session assessment tool that may support longitudinal evaluation of hemodynamic tolerance and future dialysis prescription adjustment.

Hemoglobin and hematocrit levels increase during dialysis because of hemoconcentration caused by plasma volume reduction. This physiological principle has been used for decades to estimate changes in blood, plasma, and red cell volumes based on concentration changes [17]. These calculations, originally developed in experimental settings, provide a theoretical framework for estimating intravascular volume changes using simple laboratory parameters. However, despite their physiological plausibility, these formulas have not been systematically validated in clinical populations undergoing hemodialysis. The key physiological and methodological concepts underlying intradialytic blood volume assessment are summarized in Table 1.

Although the relationship between hemoconcentration and blood volume changes has been recognized for decades, its translation into a simple, clinically applicable tool for routine hemodialysis practice has not been systematically evaluated [15,18]. Given that continuous blood volume monitoring requires dedicated technology and is not universally available or consistently used, a hemoglobin-based post-session approach may provide a pragmatic method for estimating intradialytic blood volume reduction using routinely available laboratory data. Therefore, the aim of the present study was to evaluate the agreement between hemoglobin-based estimation of intradialytic blood volume reduction and machine-based blood volume monitoring during routine hemodialysis sessions.

## 2. Materials and Methods

### 2.1. Study Design

This prospective single-center observational study was conducted in the hemodialysis unit of the Department of Nephrology and Hypertension at Barzilai University Medical Center, Ashkelon, Israel. The study was designed to evaluate the agreement between a hemoglobin-based estimation of intradialytic blood volume reduction and machine-based blood volume monitoring during routine hemodialysis treatments.

The study protocol was approved by the institutional Helsinki Committee of Barzilai University Medical Center. All study procedures were conducted in accordance with institutional guidelines and the principles of the Declaration of Helsinki (approval 0078-22-BRZ from 13 November 2022). All participants provided written informed consent prior to enrollment.

### 2.2. Study Population

Adult patients undergoing maintenance hemodialysis for at least 30 days were eligible for inclusion. This criterion was selected to ensure inclusion of clinically stable maintenance hemodialysis patients and to avoid potential variability related to the initiation phase of dialysis treatment, during which rapid changes in volume status, dialysis prescription, and hemodynamic adaptation commonly occur. Additional inclusion criteria included the ability to provide informed consent, the ability to be weighed before and after dialysis, and hemodynamic stability at the start of dialysis (defined as systolic blood pressure ≥100 mm Hg). Exclusion criteria included patients receiving acute or temporary dialysis, hospitalization at the time of study participation, active infection, or active bleeding.

The unit of analysis was the dialysis session. Multiple dialysis sessions per patient were included in the analysis, with up to 10 sessions obtained per participant. Hemodialysis sessions were included if complete pre- and post-dialysis laboratory measurements and machine-based blood volume monitoring data were available. Because repeated within-patient measurements may not be statistically independent, statistical associations and variance estimates should be interpreted in the context of clustered session-level observations.

### 2.3. Hemoglobin-Based Blood Volume Estimation

Relative blood volume reduction was estimated using changes in hemoglobin concentration measured before and after dialysis according to the following formula:

BV reduction (%) = (1 − [Hb_pre/Hb_post]) × 100

where Hb_pre and Hb_post represent pre- and post-dialysis hemoglobin concentrations, respectively.

Hemoglobin values were used as the primary parameter for calculation of formula-based blood volume reduction. Hematocrit measurements were recorded as complementary laboratory parameters but were not incorporated into the blood volume calculation model. Hemoglobin was selected because integrated blood volume monitoring systems are themselves based predominantly on hemoglobin-related hemoconcentration measurements within the extracorporeal circuit. The same hemoglobin-based formula was applied uniformly across all dialysis sessions included in the analysis.

### 2.4. Dialysis Procedures and Blood Volume Monitoring

Hemodialysis treatments were performed according to standard clinical practice protocols. Blood volume monitoring was performed using the integrated relative blood volume monitoring system (BVM module) incorporated into the Artis Physio dialysis machine (Baxter International Inc., Deerfield, IL, USA). The monitoring system estimates relative blood volume changes by continuously tracking hemoglobin-related optical density changes within the extracorporeal blood circuit and calculating proportional intravascular volume reduction relative to baseline measurements obtained at dialysis initiation.

For the present analysis, the percent blood volume reduction displayed by the dialysis machine at the end of the dialysis session, prior to blood reinfusion, was recorded and used as the reference method.

Both the machine-based monitoring system and the hemoglobin-based formula rely on hemoconcentration-related principles; therefore, the present study evaluates agreement between two indirect methods for estimating relative intradialytic blood volume reduction rather than accuracy against an absolute gold standard.

### 2.5. Blood Sampling and Laboratory Measurements

During each hemodialysis session, complete blood count samples were obtained immediately before and after dialysis. Blood samples for hemoglobin and hematocrit measurements were obtained from the arterial dialysis line immediately before initiation of dialysis and immediately at the end of the dialysis session prior to saline reinfusion. Samples were analyzed using the hospital central laboratory according to standard clinical laboratory procedures. All hemoglobin measurements were performed using the same institutional laboratory platform and standardized laboratory protocols throughout the study period. Additional clinical data collected for each session included pre- and post-dialysis body weight, blood pressure measurements, and intradialytic complications such as hypotension, clotting of the extracorporeal circuit, and post-dialysis bleeding.

### 2.6. Statistical Analysis

Continuous variables are presented as mean ± standard deviation (SD) or median with interquartile range (IQR), as appropriate. Categorical variables are presented as counts and percentages. Pearson correlation analysis was performed to evaluate the overall linear association between formula-based and machine-measured blood volume reduction values. Pearson correlation was selected because the analyzed continuous variables demonstrated approximately linear relationships and no marked deviation from normal distribution on visual inspection. Because the primary objective was to evaluate linear association between methods, Spearman correlation analysis was not considered necessary and was therefore not routinely performed. Agreement between methods was evaluated using Bland–Altman analysis, including calculation of mean bias and 95% limits of agreement. In addition, predefined agreement thresholds were evaluated by calculating the proportion of dialysis sessions in which the absolute difference between methods was within ±1%, ±2%, ±3%, ±5%, ±7%, and ±10%.

Agreement analyses, including Bland–Altman plots and predefined agreement thresholds, were considered the primary analyses for method comparison. A pragmatic prespecified agreement criterion of ≥70% of measurements within an absolute difference of ±5% was selected a priori to provide a clinically interpretable benchmark for method comparison. This threshold was not intended to define interchangeability between methods.

Exploratory linear regression analysis was performed to characterize the relationship between methods and to evaluate the potential for calibration of formula-based estimates relative to machine-derived measurements. Receiver operating characteristic (ROC) analyses were performed as exploratory secondary analyses to evaluate the ability of the formula-based estimates to identify dialysis sessions characterized by relatively large machine-measured blood volume reductions. Thresholds of ≥6%, ≥8%, ≥10%, and ≥12% machine-measured blood volume reduction were evaluated. These thresholds were selected to explore a physiologically relevant range of intradialytic blood volume changes commonly encountered during routine hemodialysis sessions and were intended for exploratory analysis rather than formal clinical outcome classification. For the ≥8% blood volume reduction threshold, sensitivity, specificity, positive predictive value (PPV), and negative predictive value (NPV) were calculated.

Additional exploratory sensitivity analysis, hematocrit-based blood volume reduction, was calculated using the same hemoconcentration principle and compared with machine-measured blood volume reduction.

No formal a priori sample size calculation was performed. The study was designed as a prospective exploratory method-comparison study. However, the final sample of 187 dialysis sessions provides reasonable precision for estimating the predefined agreement proportion. Assuming an expected agreement rate of approximately 70% within ±5%, a sample of 165 sessions would estimate this proportion with a precision of approximately ±7% using a 95% confidence interval. Therefore, the analyzed sample of 187 sessions was considered adequate for exploratory session-level agreement analysis. This precision estimate does not fully account for within-patient clustering due to repeated dialysis sessions and should therefore be interpreted as an approximate session-level justification.

All statistical analyses were performed using Python version 3.13.5 with the pandas and NumPy libraries version 2.3.5. A *p*-value < 0.05 was considered statistically significant.

## 3. Results

### 3.1. Study Sample

A total of 208 hemodialysis sessions from 25 patients were screened for inclusion. Each patient contributed up to 10 dialysis sessions, although only sessions with complete paired measurements were included in the final analysis. After exclusion of sessions with incomplete data for either the formula-based or machine-based blood volume measurement, 187 dialysis sessions were included in the final analysis. Dialysis-related treatment parameters of the analyzed hemodialysis sessions are summarized in Table 2. The most common causes of end-stage kidney disease were diabetic kidney disease (65%), cystic kidney diseases (10%), glomerular diseases (10%), nephrosclerosis (5%), and other or unknown etiologies (10%).

### 3.2. Association Between Formula-Based and Machine-Measured Blood Volume Reduction

**Figure 1:**
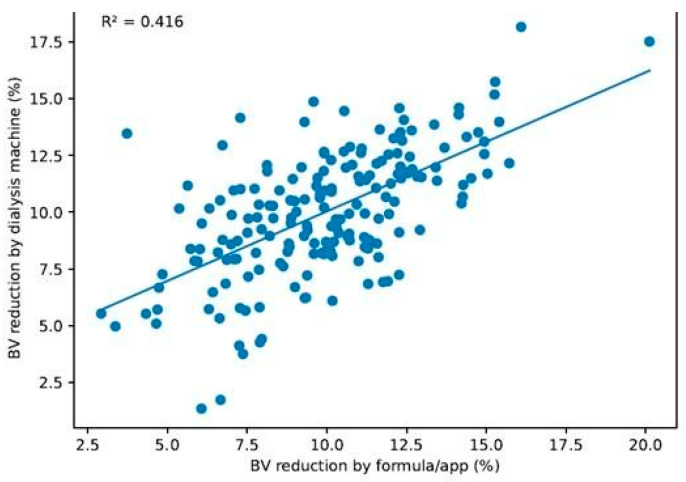
Relationship Between Formula-Based and Machine-Measured Blood Volume Reduction During Hemodialysis. Scatter plot illustrates the relationship between percent blood volume reduction during hemodialysis as estimated using a formula based on changes in hemoglobin before and after dialysis, and percent blood volume reduction measured by the dialysis machine. Each point represents a single dialysis session (n = 187). The solid line represents the linear regression between the two measurement methods. A moderate-to-strong positive linear association was observed (r = 0.645), with a coefficient of determination of R^2^ = 0.416.

Across the analyzed sessions, both methods demonstrated the same overall directional pattern of intradialytic blood volume reduction. Pearson correlation analysis demonstrated a moderate-to-strong positive linear association between formula-based estimation of blood volume reduction and machine-measured blood volume reduction (r = 0.645; Figure 1).

Higher blood volume reduction estimated by the formula was generally associated with greater blood volume reduction measured by the dialysis machine, although variability between the two methods was observed at the individual-session level.

### 3.3. Agreement Between Measurement Methods

Agreement between the two methods was assessed by calculating the absolute difference in percent blood volume reduction for each dialysis session (machine-measured BV minus formula-based BV). The proportion of measurements within predefined agreement thresholds (±1%, ±2%, ±3%, ±5%, ±7%, and ±10%) increased progressively with wider thresholds (Table 3).

**Figure 2:**
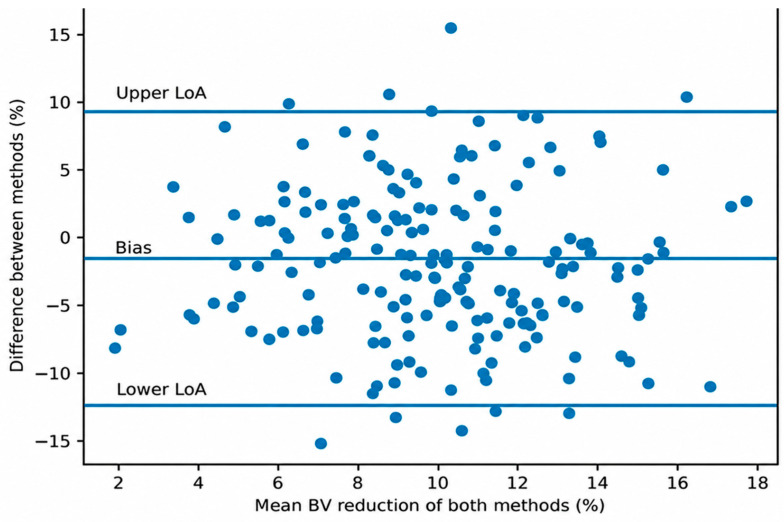
Bland-Altman analysis assessing agreement between Hemoglobin-based and machine-measured percent blood volume reduction. Bland–Altman plot shows agreement between percent blood volume reduction measured by the dialysis machine and percent blood volume reduction estimated using a formula based on changes in hemoglobin before and after hemodialysis. The *x*-axis represents the mean of the 2 measurement methods for each dialysis session, and the *y*-axis represents the difference between methods. Each point represents a single dialysis session. The central horizontal line indicates the mean bias between methods, and the upper and lower horizontal lines indicate the 95% limits of agreement. The mean bias was −1.5%, indicating that, on average, the dialysis machine measured a slightly greater blood volume reduction than the hemoglobin-based estimation, with limits of agreement ranging from −12.4% to 9.3%. LoA, limits of agreement.

The predefined pragmatic agreement criterion (≥70% of measurements within ±5%) was met, with 77.5% of measurements falling within this range. Agreement rates further increased to 88.8% and 95.2% at ±7% and ±10% thresholds, respectively. Agreement between methods was further evaluated using Bland–Altman analysis (Figure 2). Bland–Altman analysis demonstrated a mean bias of −1.5% ± 5.53%. This indicates that, on average, machine-measured blood volume reduction was 1.5 percentage points greater than the hemoglobin-based estimate. The 95% limits of agreement ranged from −12.4% to 9.3%, indicating modest average bias but substantial variability at the individual-session level.

In an exploratory sensitivity analysis, hematocrit-based estimation demonstrated a similar correlation with machine-measured blood volume reduction (r = 0.636). However, agreement within the ±5% threshold was lower for hematocrit-based estimation than for hemoglobin-based estimation (66.3% vs. 77.5%). Agreement within the ±10% threshold remained high at 91.4%, further supporting the overall physiologic consistency of the hemoconcentration-based approach while supporting the use of hemoglobin as the primary parameter in the present analysis.

### 3.4. Exploratory ROC Analysis

Exploratory ROC analyses were performed to evaluate the ability of the formula-based estimates to identify dialysis sessions characterized by relatively large machine-measured blood volume reductions. Machine-defined thresholds of ≥6%, ≥8%, ≥10%, and ≥12% blood volume reduction were used to define positive events.

**Figure 3:**
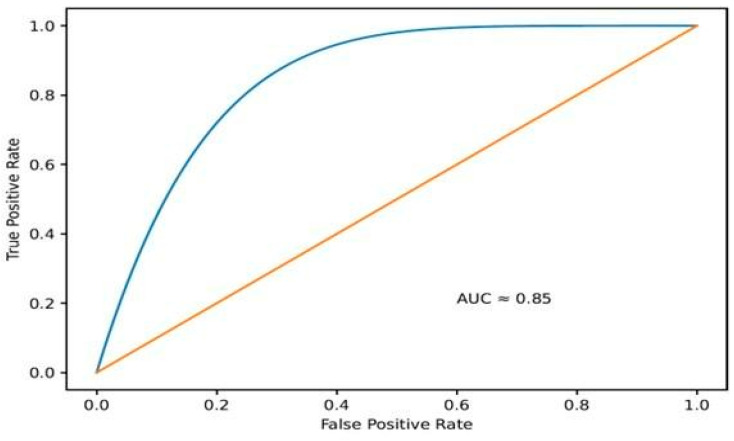
Exploratory ROC analysis for identification of sessions with large machine-measured blood volume reduction. ROC curve illustrating the exploratory ability of the hemoglobin-based formula to identify dialysis sessions characterized by larger machine-measured blood volume reduction. Machine-measured blood volume reduction was used as the reference method, with a threshold of ≥8% defined as a positive event. The blue curve represents the ROC curve of the hemoglobin-based formula, whereas the orange diagonal line represents the line of no discrimination (reference line; AUC = 0.5). The *x*-axis represents the false-positive rate (1 − specificity), and the *y*-axis represents sensitivity. The area under the curve (AUC) reflects the overall exploratory classification performance of the formula in detecting larger machine-measured blood volume reduction, with AUC values in the range of 0.84-0.87 indicating good exploratory classification performance.

The formula-based estimates were used as a continuous predictor for ROC curve construction. The area under the curve (AUC) ranged from 0.84 to 0.87 across the evaluated thresholds (Table 4 and Figure 3), indicating good exploratory classification performance relative to machine-defined blood volume reduction thresholds. For the predefined threshold of ≥8% blood volume reduction, the formula demonstrated a sensitivity of 72%, specificity of 85%, positive predictive value (PPV) of 83%, and negative predictive value (NPV) of 74%.

The formula identified most sessions with larger machine-measured blood volume reduction, while maintaining a relatively low false-positive rate. Although approximately 28% of sessions with larger machine-measured blood volume reduction were not detected by the formula, its high specificity suggests a low rate of false alerts.

### 3.5. Linear Regression Analysis

**Figure 4:**
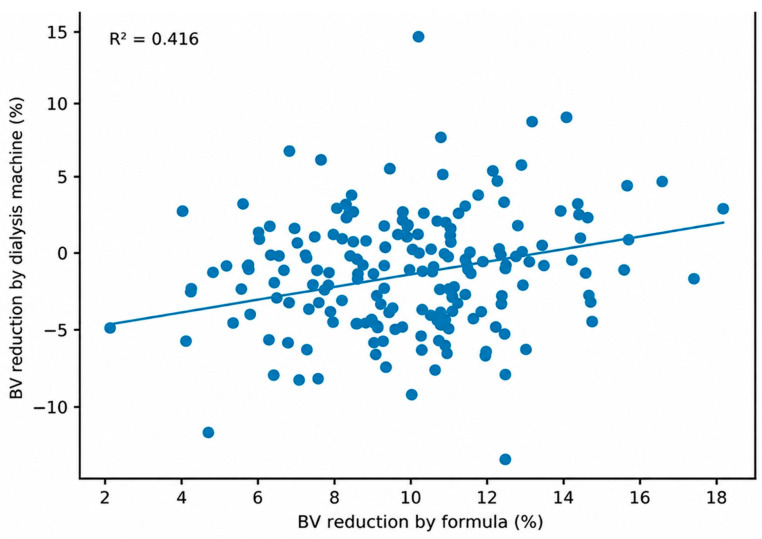
Linear regression model between Hemoglobin-based estimation and machine-measured blood volume reduction. Linear regression plot illustrates the relationship between percent blood volume reduction during hemodialysis as estimated using a formula based on changes in hemoglobin and percent blood volume reduction measured by the dialysis machine. The solid line represents the fitted linear regression model: Machine BV = −5.51 + 0.41 × Formula BV. The coefficient of determination was R^2^ = 0.416.

In univariate linear regression analysis, machine-measured blood volume reduction was modeled as the dependent variable and formula-based blood volume reduction as the independent variable (Figure 4). The exploratory regression analysis demonstrated a moderate linear association between methods, with a slope coefficient of 0.41, an intercept of −5.51, and a coefficient of determination (R^2^) of 0.416. These findings indicate that approximately 42% of the variability in machine-measured blood volume reduction was explained by the formula-based estimate. However, the modest R^2^ suggests that the 2 methods are not directly interchangeable without calibration, and that the formula provides a useful estimate of the overall trend in blood volume reduction rather than providing precise quantitative equivalence. This regression model may provide a basis for calibration of formula-based estimates to better approximate machine-measured blood volume reduction.

## 4. Discussion

In this prospective study, we evaluated the agreement between a hemoglobin-based estimation of intradialytic blood volume reduction and machine-based blood volume monitoring during hemodialysis. Across 187 dialysis sessions, we observed a moderate-to-strong correlation between methods (r = 0.645), achievement of the predefined pragmatic agreement criterion at a ±5% threshold (77.5%), and favorable exploratory classification performance for identifying sessions with larger machine-measured blood volume reduction (AUC 0.84–0.87).

These findings suggest that a formula based on routinely available laboratory parameters can capture the overall trend of intradialytic blood volume changes, although variability at the individual-session level remains.

Although a moderate-to-strong correlation was observed, correlation alone does not imply agreement. Bland–Altman analysis demonstrated a relatively small mean bias but wide limits of agreement, indicating variability between methods at the individual-session level. Accordingly, the formula should be interpreted as providing an approximation of blood volume changes rather than an exact substitute for machine-based measurements. The relatively wide limits of agreement suggest that the formula may be more suitable for identifying overall trends or recurrent patterns of intradialytic blood volume reduction rather than for precise fluid management decisions during individual dialysis sessions. The ±5% agreement threshold was selected as a pragmatic benchmark for interpretability rather than as a validated threshold for clinical interchangeability. Therefore, meeting this criterion should be interpreted as supportive of approximate agreement, not as evidence that the two methods can be used interchangeably.

The observed relationship between methods is consistent with the physiological principle of hemoconcentration during ultrafiltration. Increases in hemoglobin and hematocrit reflect reductions in plasma volume; however, this relationship is influenced by dynamic processes such as plasma refilling from the interstitial space, which varies between patients and across dialysis sessions. In clinically unstable dialysis sessions characterized by rapid ultrafiltration, impaired vascular refilling, or hemodynamic instability, changes in hemoglobin concentration may not fully reflect real-time intravascular volume changes, potentially affecting agreement between methods. These mechanisms likely contribute to the observed inter-session variability and limit precise quantitative agreement. These findings reflect session-level performance and should be interpreted in the context of repeated within-patient measurements. Hemoglobin was selected as the primary parameter because integrated blood volume monitoring systems are themselves based predominantly on hemoglobin-related hemoconcentration measurements within the extracorporeal circuit.

Correlation analysis was included to describe the overall directional association between methods, whereas regression analysis was performed primarily as an exploratory assessment of potential calibration rather than as a measure of agreement. ROC analyses were included as exploratory secondary analyses and should not be interpreted as evaluation of diagnostic accuracy against a clinical gold standard. Rather, they reflect the ability of the formula-based estimates to identify sessions characterized by larger machine-measured blood volume reductions. Because no clinical outcome measure was used, ROC-derived metrics reflect agreement with the reference method rather than prediction of patient-centered endpoints.

The blood volume reduction thresholds evaluated in the exploratory ROC analyses were not intended to represent validated clinical cutoffs for intradialytic hypotension or adverse outcomes. Rather, they were selected to explore a range of physiologically relevant blood volume reductions commonly encountered during routine dialysis treatment. Previous studies have demonstrated associations between marked intradialytic blood volume reduction and hemodynamic instability, although no universally accepted threshold currently exists [19,20]. Because intradialytic hypotension events and symptom severity were not systematically analyzed in the present study, the clinical implications of specific blood volume reduction thresholds remain uncertain.

Monitoring intradialytic blood volume is clinically relevant because marked reductions in blood volume during dialysis may be associated with intradialytic hypotension and symptoms such as weakness, dizziness, and muscle cramps. Previous studies have demonstrated that relative blood volume monitoring and hemoconcentration-based assessment approaches may support evaluation of intradialytic hemodynamic stability and fluid management during hemodialysis [21,22]. However, the proposed hemoglobin-based method is not designed to provide real-time intradialytic feedback or automated biofeedback control. Rather, its potential utility lies in post-session assessment and longitudinal evaluation of recurrent patterns of excessive intradialytic blood volume reduction.

In this context, hemoglobin-based estimation should be viewed as a practical adjunct rather than a replacement for continuous monitoring. By identifying patients or sessions with repeated large blood volume reductions, this approach may help clinicians interpret hemodynamic tolerance over time and consider subsequent adjustments in ultrafiltration targets, treatment duration, or dry weight assessment, particularly in settings where integrated blood volume monitoring is unavailable or inconsistently used. Future calibration models may further improve agreement between hemoglobin-based and machine-based estimates.

Blood volume monitoring during hemodialysis has been extensively studied and typically based on continuous optical measurements within the extracorporeal circuit [16]. While early studies suggested potential benefits for fluid management and dry weight assessment [23], subsequent randomized trials have shown mixed results regarding their impact on clinical outcomes. In the CLIMB study, a multicenter randomized trial evaluating blood volume monitoring using the Crit-Line system, no clear benefit was demonstrated compared with standard clinical monitoring [24]. These findings highlight that blood volume monitoring is best considered a complementary tool rather than a standalone determinant of clinical decision-making.

In addition, previous studies have shown that indirect biological markers, such as changes in hemoglobin or hematocrit, can be used to estimate blood volume changes [21]. The present study extends this concept by evaluating its performance in a real-world clinical setting and by framing it as a potential practical tool for routine use. As previously described, blood volume monitoring reflects only part of the complex hemodynamic changes occurring during dialysis and should be interpreted in conjunction with clinical parameters [20].

The present study addresses this gap by demonstrating that routinely available laboratory parameters can be used to estimate intradialytic blood volume changes with potentially clinically useful performance. Importantly, this approach does not require specialized equipment and may therefore expand access to blood volume assessment in settings where dedicated monitoring systems are unavailable.

Several limitations of the present study should be acknowledged. First, this was a single-center study with a relatively limited number of patients, which may restrict the generalizability of the findings. Although 187 dialysis sessions were analyzed, these measurements were derived from 25 unique patients, and repeated measurements within patients may have introduced within-patient clustering and reduced statistical independence between observations. Because standard correlation, regression, and agreement analyses assume statistical independence, variance estimates and confidence measures may have been affected by repeated observations from the same individuals.

Second, both the machine-based monitoring system and the hemoglobin-based formula represent indirect hemoconcentration-based approaches for estimating relative blood volume reduction rather than absolute measurements of intravascular volume. Therefore, the present study should be interpreted as an agreement study comparing two indirect estimation methods, rather than a study of absolute measurement accuracy.

Third, several dialysis-related and physiologic factors that may influence intradialytic hemoconcentration and blood volume dynamics, including ultrafiltration rate, dialysate sodium concentration, plasma-refilling capacity, and body weight changes, were not systematically incorporated into the present analysis.

Finally, intradialytic hypotension events and dialysis-related symptoms were not formally correlated with blood volume reduction estimates, and the study did not establish clinically validated blood volume reduction thresholds for predicting intradialytic complications.

Future multicenter studies including larger and more diverse hemodialysis populations are needed to further validate these findings. Future investigations should also evaluate whether incorporation of dialysis prescription variables and clinical outcomes, including intradialytic hypotension and dialysis intolerance, improves the clinical applicability and prognostic value of hemoglobin-based blood volume estimation.

## 5. Conclusions

A hemoglobin-based approach may provide a simple and accessible method for longitudinal assessment of intradialytic blood volume trends, demonstrating moderate agreement with machine-based monitoring and good exploratory classification performance relative to machine-defined blood volume reduction thresholds. While not interchangeable with continuous blood volume monitoring and not intended for precise individual-session fluid management, this approach may serve as a practical adjunctive tool for post-session assessment of intradialytic hemodynamic tolerance, particularly in settings where dedicated monitoring systems are unavailable. Further studies are needed to validate these findings and to evaluate their association with clinical outcomes and dialysis-related complications.

## Figures and Tables

**Table 1:** Conceptual overview of intradialytic blood volume assessment methods.

Concept	Definition	Clinical Relevance
Relative blood volume reduction	Reduction in intravascular blood volume during hemodialysis, primarily due to ultrafiltration and plasma volume removal.	Reflects intradialytic volume stress and may be associated with hemodynamic instability.
Hemoconcentration	Increase in hemoglobin concentration during dialysis as plasma volume decreases while red cell mass remains relatively unchanged.	Provides the physiological basis for estimating blood volume changes from hemoglobin measurements.
Machine-based blood volume monitoring	Continuous estimation of relative blood volume changes using optical hemoglobin-related measurements within the extracorporeal circuit.	Provides real-time intradialytic trends but requires dedicated monitoring technology.
Hemoglobin-based estimation	Post-session calculation of blood volume reduction using pre- and post-dialysis hemoglobin values.	Provides a simple retrospective estimate using routinely available laboratory data.

Conceptual overview of the principal physiological and monitoring concepts related to intradialytic blood volume assessment during hemodialysis. The table summarizes the definitions, physiological basis, and clinical relevance of machine-based blood volume monitoring and hemoglobin-based estimation approaches evaluated in the present study.

**Table 2:** Dialysis Sessions Related Parameters.

Analyzed dialysis sessions, n	187
Dialysis duration, hours, mean ± SD	3.77 ± 1.17
Planned ultrafiltration volume, L, mean ± SD	2.52 ± 1.17
Actual weight reduction, Kg, mean ± SD	2.06 ± 1.18
Dialyzer type, FX100, n (%)	187 (100)
Dialysate potassium, n (%)	
Potassium 2 mEq/L	145 (77.5)
Potassium 3 mEq/L	24 (12.8)
Potassium 4 mEq/L	18 (9.6)
Dialysate calcium, n (%)	
Calcium 1.25 mEq/L	164 (87.7)
Calcium 1.5 mEq/L	23 (12.3%)
Intradialytic hypotension recorded, n (%)	5 (2.7)

Dialysis-related parameters are presented at the session level for the 187 analyzed hemodialysis sessions. Continuous variables are presented as mean ± standard deviation, and categorical variables are presented as number and percentage. mEq/L, milliequivalents per liter; L, liters; Kg, kilograms.

**Table 3:** Agreement rates between hemoglobin-based and machine-measured blood volume reduction across predefined thresholds.

Absolute Difference Threshold (%)	Agreement (%)
±1%	16.6
±2%	34.2
±3%	48.7
±5%	77.5
±7%	88.8
±10%	95.2

Agreement was defined as the proportion of dialysis sessions in which the absolute difference between methods was within the specified threshold.

**Table 4:** Area under the ROC curve (AUC) for different thresholds of blood volume reduction.

Blood Volume Reduction Threshold (%)	Area Under Curve (AUC)
≥6%	0.86
≥8%	0.85
≥10%	0.87
≥12%	0.84

AUC indicates the exploratory classification performance relative to machine-defined thresholds of the hemoglobin-based estimation for identifying sessions with machine-defined blood volume reduction at each threshold.

## Data Availability

The data that support the findings of this study are not publicly available due to their containing information that could compromise the privacy of research participants but are available from NLI the corresponding author.
